# Fungal endophyte-derived *Fritillaria unibracteata* var. *wabuensis*: diversity, antioxidant capacities *in vitro* and relations to phenolic, flavonoid or saponin compounds

**DOI:** 10.1038/srep42008

**Published:** 2017-02-06

**Authors:** Feng Pan, Tian-Jiao Su, Shi-Mei Cai, Wei Wu

**Affiliations:** 1 Department of Medicinal Plants, Agronomy College, Sichuan Agricultural University, No. 211, Huimin Rd, Wenjiang District, Chengdu 611130, Sichuan, PR China

## Abstract

Diverse fungal endophytes are rich fungal resources for the production of an enormous quantity of natural products. In the present study, 53 fungal endophytes were isolated from the bulbs of *Fritillaria unibracteata* var. *wabuensis* (FUW). Of these, 49 strains were identified and grouped into 17 different taxa, and priority was conferred to the *Fusarium* genus. All fungal fermented filtrates displayed antioxidant activities. The DPPH activity, total antioxidant capacities (ABTS), reduction power (FRAP), total phenolic content (TPC), total flavonoid content (TFC) and total saponin content (TSC) were evaluated using petroleum ether, ethyl acetate, n-butyl alcohol and ethanol fractions extracted from five representative fungal cultures. The last three fractions showed more potent antioxidant activity than the first fraction. Significant positive correlations were found between the compositions (TPC, TFC and TSC) and antioxidant capacities (DPPH, ABTS and FRAP). In addition, multifarious natural antioxidant components were identified from the fungal extracts, including gallic acid, rutin, phlorizin, 2,4-di-tert-butylphenol and 2,6-di-tert-butyl hydroquinone; these were determined preliminarily by TLC-bioautography, HPLC and GC-MS analysis. This study showed abundant fungal resources in FUW. Phenolics, flavonoids and saponins are crucial bioactive constituents in these abundant fungal endophytes and can be viewed as new potential antioxidant resources.

Oxygen free radicals or, more generally, reactive oxygen species (ROS), are continuously produced as byproducts of aerobic metabolism in plants and humans^1^. ROS are crucial and beneficial to living systems^1^,^2^ and have roles as signalling molecules in the regulation of numerous biological processes^3^. In addition, ROS play an indispensable role in plant defence response under various biotic and abiotic stresses, such as pathogen, hyperthermia and high radiometry stress^4^,^5^. However, excessive ROS can damage cellular lipids, proteins, cells, tissues and organs and can inhibit the normal function of DNA, which can induce diseases such as cancer, male factor infertility, heart disease, Alzheimer’s disease, and ageing over the human life cycle^6^,^7^,^8^. In plants, excessive ROS can cause a great deal of plant cell damage and lead to toxicity^4^. Therefore, antioxidant radical scavenging is necessary for humans and/or plants when increasing ROS escape redox regulation and exceed the upper limit of the organism.

Fungal endophytes that invade or live inside plant tissues without causing harm to hosts^9^ are a class of increasingly important microbial resources. It is estimated that there are approximately 1 million endophytes in plants^10^ that produce various bioactive substances under limited growth space, specific natural environments and particular lifestyles. A large number of novel or valuable active constituents can be acquired from fungal endophytes^11^. For example, indole diketopiperazines from fungal endophytes can inhibit the growth of breast cancer cells^12^. Approximately half of the newly discovered fungal metabolites were isolated from endophytic strains in the decade from 2002 to 2012, and fungal endophytes may possess the potential to produce bioactive compounds, including antioxidant components^13^. Therefore, it is highly important to explore the secrets of fungal endophytes in relation to their antioxidant components.

Bulbus *Fritillariae* cirrhosae (BFC) is a valuable and expensive Chinese herb that has been used in traditional Chinese medicine (TCM) for more than 2000 years. It continues to be one of the most widely used remedies in TCM^14^,^15^,^16^. *Fritillaria unibracteata* Hsiao et K.C. Hsia var. *wabuensis* (S.Y. Tang et S.C.Yue) Z.D. Liu, S. Wang et S.C. Chen (*F. unibracteata* var. *wabuensis*, FUW) was recorded in the 2010 edition of China Pharmacopoeia as one of the sources of BFC (Pharmacopoeia of the People’s Republic of China). It is considered to be one of the original sources of BFC and the most appropriate for cultivation. Cultivated FUW is primarily distributed at altitudes of 2500 to 3000 m in the Qinghai-Tibet Plateau, where it experiences high ultraviolet (UV) radiation and cold stress. The plant´s environment may stimulate fungal endophytes to synthesise antioxidant compounds to adapt or respond to biotic and abiotic stresses^17^.

Phenolic acids, flavonoids, tannins and other compounds have been widely studied as the primary free radical scavenging molecules in many medicinal plants^17^,^18^. Phenolic compounds are reportedly the primary antioxidant components of secondary metabolites from fungal endophytes with antioxidative activity^19^,^20^. However, most reports have only explored the relations between a single endophytic fungus and its phenolic compounds. It is still unknown whether there is a similar rule in the fungal endophytic community for one type of plant. Therefore, the diversity of the fungal endophytic community derived from FUW bulbs was evaluated in this paper. The antioxidant capacities of different organic solvent-extracted fractions from partially screened fungal endophytes were evaluated using FRAP, DPPH and ABTS assays. The total contents of phenolic, flavonoid and saponin compounds were detected by ultra-violet spectroscopy, and correlations with the corresponding antioxidant capacities were analysed. Our results showed that phenolic, flavonoid and saponin compounds, particularly phenolics and flavonoids, play a conspicuous role in the antioxidation activities of extracts from multiple fungal endophytes. The TLC, HPLC and GC/MS analyses also preliminarily confirmed diverse phenolic, flavonoid and saponin antioxidants.

## Results and Analysis

### Fungal diversity

**Figure 1:**
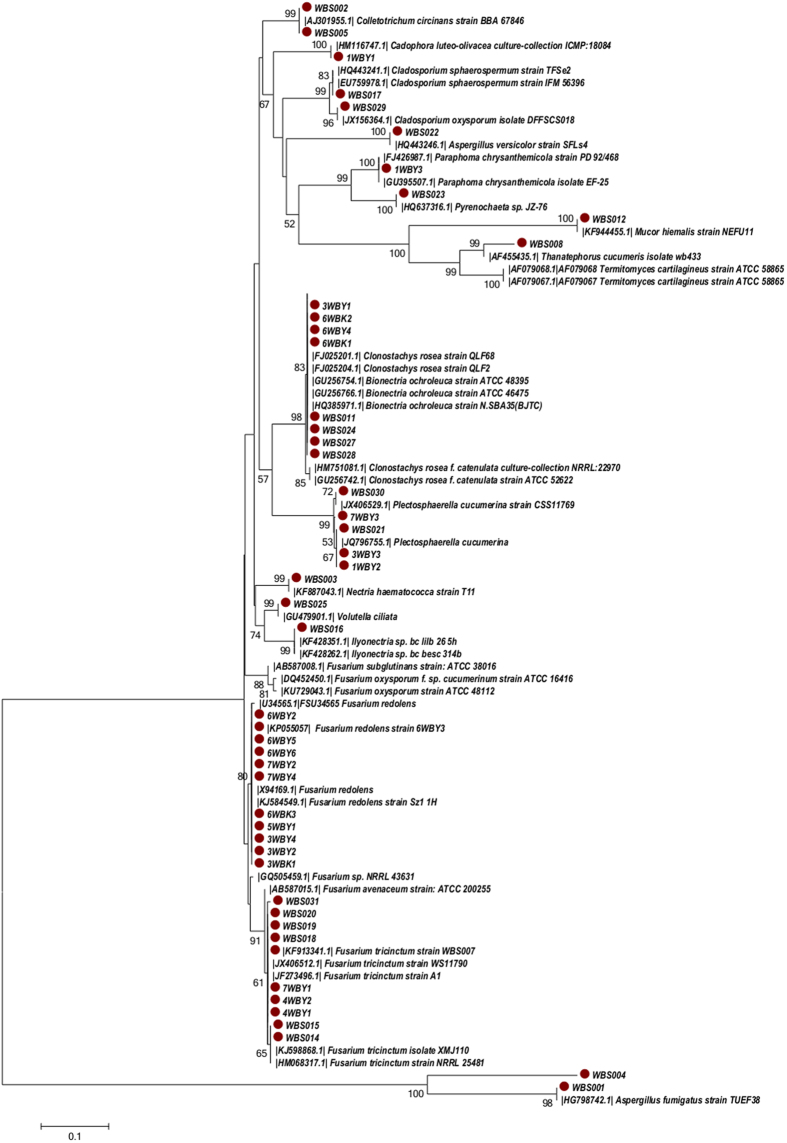
Neighbour-joining method analysis of ITS1-5.8S rDNA-ITS2 sequences from the endophytic fungi of *Fritillaria unibracteata* var. *wabuensis*. The tree was derived from the sequences of 49 endophytic fungi (marked with symbols) and 41 sequences retrieved from GenBank. The percentage of replicate trees in which associated taxa were clustered together in the bootstrap test (1000 replicates, values below 50% are not shown) are shown next to the branches. Phylogeny analyses were conducted in MEGA5.1 software.

Fifty-three strains of morphologically distinct fungal endophytes were isolated from FUW. The sequences of two isolates (strains WBS007 and 6WBY3) from a former study were included with the 47 sequences of isolates from this study^11^. The amplified ITS1-5.8S rDNA-ITS2 regions of the isolates (47 isolates) were sequenced and studied by BLAST-searching the GenBank database. The closest ITS region sequences were selected for further analysis, and the closest identified matching strains are listed in Table 1. All nucleotide sequences of these isolates were no less than 99% similar to the closest match from the nucleotide database, except for WBS008, which had 91% similarity (Table 1). The phylogenetic relations of these isolates with their related standard fungi strains in the NCBI GenBank database were analysed by MEGA 5.1 software using the neighbour-joining method, and the results are shown in Fig. 1. The forty-nine isolates were grouped into 17 different taxa (Fig. 1) that exhibited abundant biodiversity. The most common taxa were *Fusarium redolens* and *Fusarium tricinctum* (11 isolates and 10 isolates, respectively), followed by *Clonostachys rosea* (teleomorph, *Bionectria ochroleuca*) and *Plectosphaerella cucumerina* (8 isolates and 5 isolates, respectively). WBS008 was ascribed to *Thanatephorus* sp. according to the phylogenetic analysis (Fig. 1).

### Antioxidant activity screening

Abundant antioxidant compounds are produced by fungal endophytes^21^. Each compound has a different antioxidant efficacy, which is very difficult to measure with a single antioxidant assay^22^. Several methods, including the FRAP assay and the DPPH radical scavenging assay, were used to evaluate the antioxidant activities of the filtrates of the fungal endophytes.

The antioxidant activity screening results are shown in Table 2. The filtrates of fungal endophytes all showed antioxidant activities by both the FRAP and DPPH assay. The antioxidant values ranged widely, from 84.60 ± 1.56 to 1104.44 ± 25.17 reducing power and 6.88 ± 0.14% to 107.32 ± 8.91% DPPH radical inhibition (Table 2). The results showed that 30 isolates (62.0% of the total) had more than 550 μM FRAP activity (half the maximum value of FRAP in the FRAP assay). In two isolates (6WBY2 and 6WBK3) from the *Fusarium* genus and one (WBS026) unidentified isolate, the filtrate showed FRAP activities of greater than 1 000 μM. In addition, 20 isolates (39.2% of the total) showed more than 50% DPPH radical inhibition. Among these isolates, 7 isolates showed more than 90% inhibition, including one isolate (WBS027) from the *Bionectria* genus, one (7WBY2) from *Fusarium* and one (WBS013) that was unidentified, the filtrate of which showed near-total DPPH radical inhibition (≈100% inhibition; Table 2). This finding indicated that fungal endophytes from FUW are a potential antioxidant resource worthy of further study. However, the FRAP and DPPH values were difficult to compare directly because of their dimensional quantity. These fuzzy membership methods had the same value for x, and all had the same membership when 1 was used according to the method described by Chen^23^. Nineteen isolates (37.2% of the total) exhibited a value above 1 for the sum of the degree of Y_FRAP_ and Y_DPPH_ membership. Five isolates (4WBY1, 6WBY3, 7WBY2, WBS019 and WBS020) were selected randomly from the 19 isolates for further study, but priority was given to fungi with good growth states and epigenetic stability on PDB.

### *In vitro* antioxidant activities of different fractions from fungal endophytes

Three different methods, namely the FRAP assay, the DPPH radical scavenging assay and the ABTS radical scavenging assay, were used to evaluate the antioxidant activities of the petroleum ether (30–60 °C, PE) fractions, ethyl acetate (EA) fractions, n-butyl alcohol (BA) fractions and absolute ethanol (ET) fractions extracted from the 5 isolates.

#### DPPH and ABTS radical scavenging activity

The antioxidant capacities of all extracts were evaluated for their ability to scavenge the two commonly used synthetic radicals DPPH and ABTS. Trolox and Vc were chosen as the positive controls.

**Figure 2:**
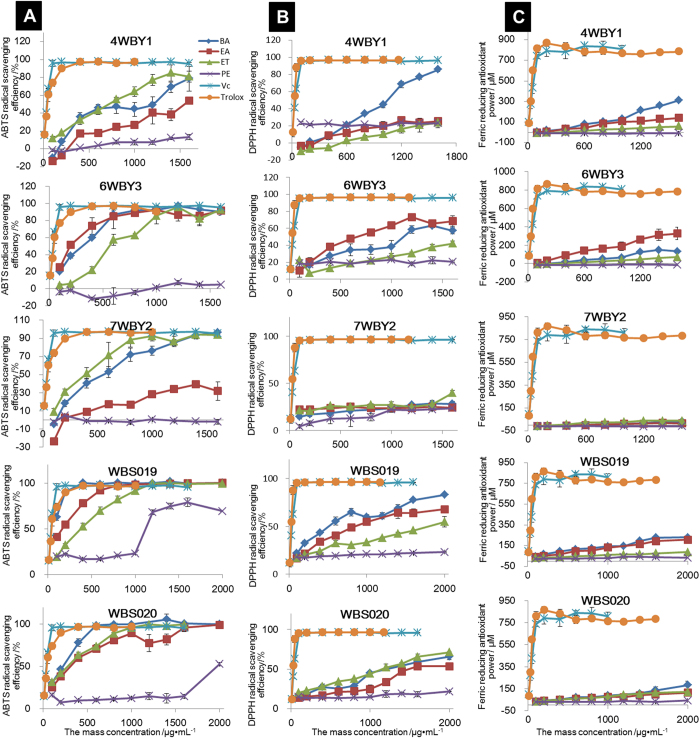
Antioxidant assays of petroleum ether (PE, 30–60 °C), ethyl acetate (EA), n-butyl alcohol (BA) and ethanol absolute (ET) fractions extracted from five representative fungal endophytes (4WBY1, 6WBY3, 7WBY2, WBS019 and WBS020). (**A**) Total antioxidant capacities (ABTS assay), (**B**) DPPH free radical-scavenging activity and (**C**) ferric-reducing antioxidant power.

As seen in Fig. 2A and Supplementary Table S1, all samples except the PE fractions from the five fungal endophytes exhibited obvious ABTS free radical scavenging activity in a concentration-dependent pattern at all concentrations. In particular, at 1.00 mg·mL^−1^, half of the fractions (the EA, BA and ET fractions from isolate 6WBY3; the BA and ET fractions from isolate 7WBY2; the EA, BA, and ET fractions from WBS019; and the EA, BA and ET fractions from WBS020) exhibited a near 100% scavenging rate of hydroxyl radicals, with a range of IC_50 ABTS_ from 69.92 to 625.77 μg·mL^−1^. The BA fraction from WBS019 exhibited the strongest scavenging activity, which increased significantly with concentration (IC_50 ABTS_: 69.92 μg·mL^−1^), among those fractions. This fraction exceeded 100% ABTS radical scavenging efficiency at 0.40 mg·mL^−1^ and approached the activity of Trolox and Vc at 0.10 to 0.40 mg·mL^−1^. In contrast, the ABTS free radical scavenging ability of PE fractions from all isolates was always less than 25% from 0.10 to 1.00 mg·mL^−1^, indicating poor antioxidant activity.

The capacities of 20 extract fractions assayed to scavenge the DPPH radical in comparison with the antioxidants Trolox and Vc are shown in Fig. 2B and Supplementary Table S1. The scavenging ability of PE fractions was significantly lower than that of other tested fractions, Trolox and Vc (control). On the contrary, EA and BA fractions showed relatively higher scavenged activities than the other fractions in the DPPH assay. For example, at 1.6 mg/mL, the DPPH radical-scavenging activities of the EA fractions from strains 6WBY3 and WBS019 were 68.45 ± 6.08% and 63.89 ± 4.20%, respectively, and that of the BA fractions from strains 4WBY1 and WBS019 were 76.70 ± 2.39% and 77.73 ± 1.65%, respectively. All fractions had lower DPPH radical-scavenging activities than the positive controls (Vc and Trolox), especially extracts from strain 7WBY2. Supplementary Table S1 also shows the same results. PE fractions had the lowest values of IC_50 DPPH_ among all fractions and standard controls (Vc and Trolox) except the EA fraction from 7WBY2, while the BA fraction from WBS019 and the EA fraction from 6WBY3 had higher values of IC_50 DPPH_ than other fractions at 696.12 and 834.30 μg·mL^−1^, respectively.

In addition, the ABTS radical scavenging capacity was generally stronger than the capacity for DPPH (Fig. 2A,B). However, there was extremely significant correlation between ABTS and DPPH radical scavenging capacities (*P* < 0.01) (Table 3).

#### Reduction power (FRAP assay)

The total reduction power was measured using the ferric ion-reducing antioxidant power (FRAP) assay. The reducing power of all fractions and standard controls is shown in Fig. 2C. The reducing power of all fractions was notable at all tested concentrations and positively correlated with increasing concentration up to 1.60 mg·mL^−1^. The FRAP values of the BA fraction from strain 4WBY1 (308.26 ± 13.66 μM at 1.60 mg·mL^−1^) and the EA fraction from strain 6WBY3 (327.35 ± 67.48 μM at 1.60 mg·mL^−1^) were distinctly higher than that of the other fractions. In contrast, the PE fractions from the five strains had the lowest activity among all samples. In terms of a single strain, the ferric reducing power of the extractions from strain 7WBY2 was lower than that of the other strains. Additionally, the reducing power of fractions was generally lower than the scavenging activity (DPPH and ABTS free radical) (Fig. 2C, Table S1).

### Quantitative analysis of phytochemicals

**Figure 3:**
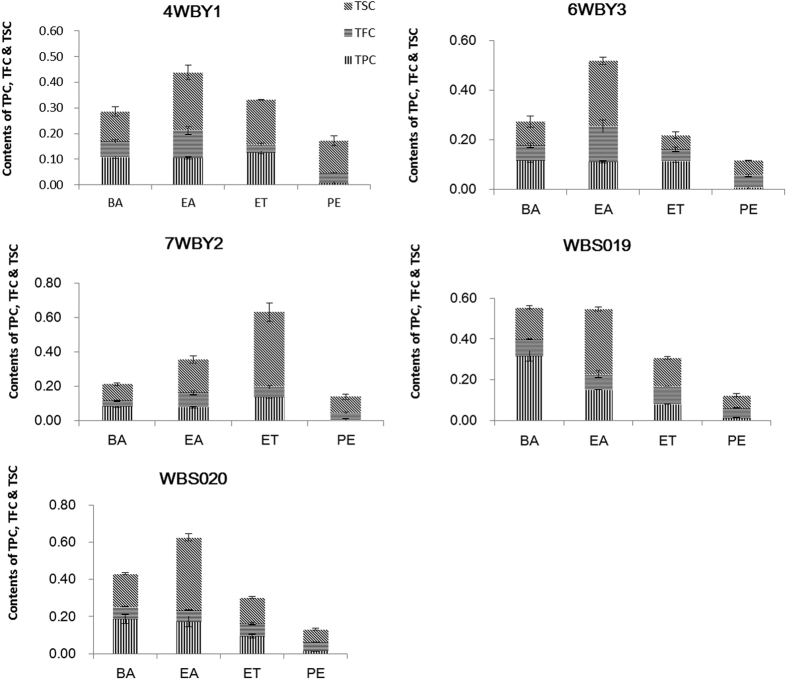
The total phenolic (TPC), total flavonoid (TFC) and total saponin (TSC) contents in the petroleum ether (PE, 30–60 °C), ethyl acetate (EA), n-butyl alcohol (BA) and ethanol absolute (ET) extracts from five fungal endophyte isolates (4WBY1, 6WBY3, 7WBY2, WBS019 and WBS020). The values are the mean ± SD (n = 3).

The total phenolic content (TPC), total flavonoid content (TFC) and total saponin content (TSC) in the PE, EA, BA and ET fractions from the five fungal endophytes (4WBY1, 6WBY3, 7WBY2, WBS019 and WBS020) are shown in Fig. 3. The TPC, TFC and TSC were expressed in gallic acid equivalents (GAE) per mg of extract, rutin equivalents in mg per mg of extract and oleanolic acid equivalents (OAEs) per mg of extract, respectively. Figure 3 shows that the PE fraction in all tested fungal endophytes contained the least TPC, TFC and TSC and possessed the weakest antioxidant capacity. Additionally, the endophytic isolates with the highest total antioxidant activities normally contained high levels of TPC, TFC and TSC constituents.

Statistically significant relations were observed between the components (TPC, TFC and TSC) and their antioxidant capacities (DPPH, ABTS and FRAP) (Table 3 and Supplementary Fig. S1). Table 3 shows that the correlation coefficients between the TPC and free radical-scavenging capacities of DPPH and ABTS were statistically significant at the *P* < 0.01 level; the same was true between the TFC and the free radical-scavenging capacities of ABTS and reduction power (FRAP value). There was statistical significance at the *P* < 0.05 level between the TSC and free radical-scavenging capacity of ABTS. Thus saponins, especially phenolics and flavonoids, played an important, but varied, role in the antioxidant reactions of the fungal endophytes.

### Qualitative analysis of phytochemicals

#### TLC-Bioautography analysis

**Figure 4:**
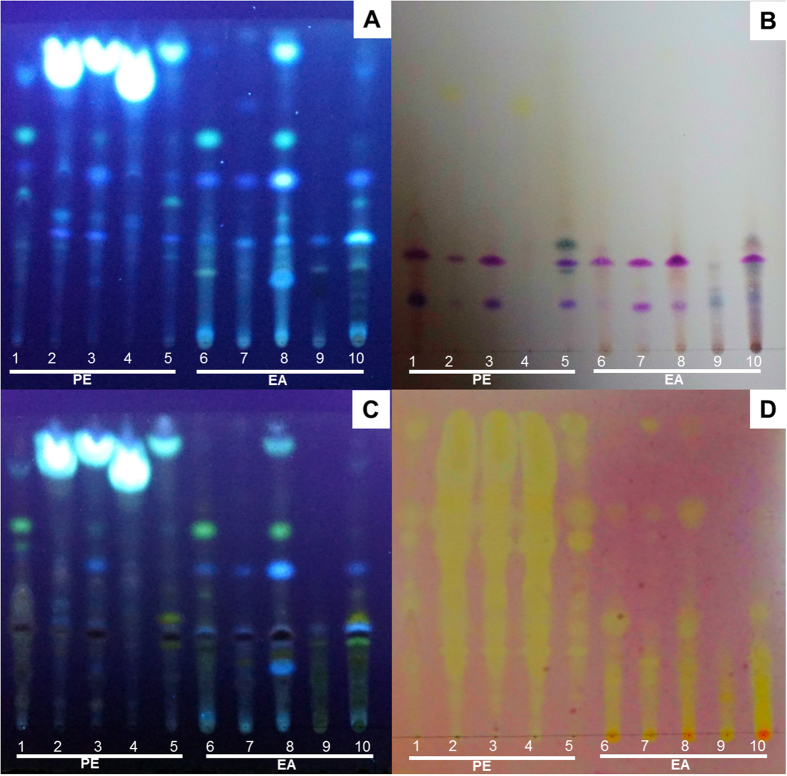
TLC photography of fungal endophytic extractions. The petroleum ether (PE, 30–60 °C) and ethyl acetate (EA) fractions extracted from strains 4WBY1 (1 and 6), 6WBY3 (2 and 7), 7WBY2 (3 and 8), WBS019 (4 and 9) and WBS020 (5 and 10). These fractions were developed in a preselected solvent system containing trichloromethane/toluene/ethanol/formic acid (4:4:0.5:0.1, by volume) and visualised using several methods, namely, ultraviolet lamps at 365 nm (**A**), heating for 3 min at 105 °C after spraying with vanillin sulphuric acid (**B**), ultraviolet lamps emitting at 365 nm after spraying with 2% aluminium chloride solution (**C**) and 0.04 mg·mL^−1^ DPPH in ethanol (**D**).

Direct bioautography combined with thin layer chromatographic (TLC) separation is a rapid and sensitive method for the detection of antioxidant compounds. To directly observe the diversity of metabolic composition and antioxidant activities, fractions were subjected to TLC-bioautography analysis. Of the various mobile phases tested, trichloromethane/toluene/ethanol/formic acid (4:4:0.5:0.1, by volume) gave the best resolution of compounds from the PE and EA fractions (R_*f*_ = 0.05–0.95). However, the BA and ET fractions were poorly resolved under multiple conditions. Therefore, the TLC-Bioautography analysis of PE and EA fractions were selected in this report. The presence of strongly adsorbing sites in Fig. 4A showed that many compounds from the PE and EA fractions were separated with ultraviolet lamps emitting at 365 nm. The PE fraction from fungal endophytes on Silica gel G plates had higher retention factors (*R*_*f*_ from 0.4 to 1.0) than those of the EA fraction; in fact, some compounds from the EA fraction remained at the baseline. The results suggested that the mobile phase showed stronger developing power for compounds from PE than EA. After heating for 3 min at 105 °C after spraying with vanillin sulphuric acid, multiple tinctorial spots with *R*_*f*_ < 0.5 appeared, suggesting the presence of saponin, phenols, sterols or terpenoids. (Fig. 4B). Further, two spots (*R*_*f*_ = 0.28 and *R*_*f*_ = 0.15) were components common to the PE and EA fractions of 4WBY1, 6WBY3, 7WBY2 and WBS020 (Fig. 4B). In addition, when visualised using an aluminium chloride solution, multiple yellow or green-yellow florescence under UV_365_ nm revealed the existence of flavonoid compounds in other fractions (but not the PE fractions of 6WBY3, 7WBY2 and WBS019) (Fig. 4C). Compared with the TLC plate sprayed with DPPH solution (Fig. 4D), most of the adsorbing sites in Fig. 4A exhibited good antioxidant activity. Figure 4D shows that PE fraction chromatography revealed more yellow spots than EA fraction chromatography. This may be because most PE fractions were easier to spread on the silica gel G plate under the solvent system used. The baseline of all EA fractions ranged from deep yellow to orange, suggesting that antioxidant constituents were present. Most chromatographic sites, including the two common component spots (*R*_*f*_ = 0.28 and *R*_*f*_ = 0.15) in Fig. 4B, indicated the interaction with DPPH free radicals based on recognisable yellow spots (Fig. 4D). In addition, all visualised flavonoids (yellow or green-yellow spots in Fig. 4C) exhibited strong DPPH radical-scavenging activity according to the emergence of corresponding yellow spots (Fig. 4D). These results indicated that the fungal endophytes from FUW produce various metabolic products including multiple flavonoid compounds, saponin, phenols, sterols or terpenoids, which could remove DPPH free radicals.

#### HPLC analysis

**Figure 5:**
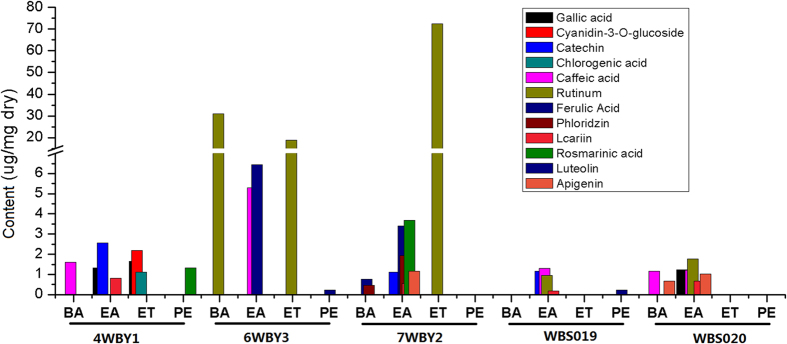
The phenolic antioxidant contents of petroleum ether (PE, 30–60 °C), ethyl acetate (EA), n-butyl alcohol (BA) and ethanol absolute (ET) fractions extracted from five representative fungal endophytes (4WBY1, 6WBY3, 7WBY2, WBS019 and WBS020).

The results of TLC-bioautography analysis certified that there were abundant antioxidants in the extracts of the fungal endophytes. The HPLC method performed in this study was aimed at a preliminary affirmation of the existence of partial common phenolic antioxidants. The chromatograms showed that all fungal extracts yielded multiple peaks that matched the retention time (RT) of authentic polyphenol antioxidants, despite the differences among the extract fractions and isolates (Supplementary Fig. S2a–e). For example, the chromatogram of the ethyl acetate fraction from strain 4WBY1 had three peaks matching the RTs of authentic gallic acid (1, 6.282 min), catechin (3, 9.404 min) and icariin (9, 14.963 min), and the PE fraction had one peak that matched the RT of rosmarinic acid (10, 15.695 min) (Supplementary Fig. S2a). Considering the number of peaks in different fractions from the same isolate, ethyl acetate was suited to extracting polyphenol analytes; otherwise, petroleum ether was better. Considering the number of peaks in different isolates, 7WBY2 produced the most compounds, the peaks of which coincided with the standards. In addition, according to the external standard curve method, the polyphenol antioxidant contents from the five isolates were calculated and are presented in Fig. 5. The yields of the analytes from the five isolates were at the μg·mg^−1^ dry extract level. The analyte with the maximum content was rutin, with 72.57 μg·mg^−1^ dry extract in the ET fraction from strain 7WBY2 (Fig. 5F). The range of contents for most analytes (approximately 85%) was from 0.1 to 5.0 μg·mg^−1^ dry extract. These results indicated that all 11 analytes, except for apigenin were produced by fungal endophytes from FUW, but at very low amounts.

In addition, caffeic acid (CA) and ferulic acid (FA) were detected in five fractions. The correlation analysis showed very notable relativity (*P* < 0.01) between CA and TFC and between FA and TFC/TSC (Table 3). Similarly, there was a significant correlation (*P* < 0.05) between FA and FRAP/DPPH. Although there was no significant correlation between CA and antioxidant activity, the R-values were very high between CA and FRAP (R = 0.858)/DPPH (R = 0.629). The result showed that CA and FA were the main antioxidant compounds produced by some fungal endophytes.

#### GC-MS analysis

GC-MS is applied for the separation of complex mixtures with low polarity or volatile substances on capillary columns. The increased fragmentation afforded by GC/MS allows for the identification of unknowns by searching a mass spectral database. The PE fraction had the lowest polarity and was the most volatile among all extract fractions. Therefore, GC-MS analysis was applied to the PE fraction to reveal its antioxidant compounds. The PE fractions from strains 4WBY1, 6WBY3 and 7WBY2 were applied to GC-MS. Twenty-nine, 50 and 27 components from the PE fraction of strains 4WBY1, 6WBY3 and 7WBY2 were identified using the NIST database with R_match_ values greater than 90% (Supplementary Tables S2–4). In contrast, four, five and three phenol components were identified from the PE fractions of the 4WBY1, 6WBY3 and 7WBY2 strains, respectively (Table 4). The percentage of butylated hydroxytoluene was up to 9.71% of the total content of the PE fraction from 7WBY2 using an area normalisation method.

## Methods

### Isolation of the fungal endophytes

Fungal endophytes were isolated from the fresh bulbs of FUW that had been cultivated for 1~6 years in the western Sichuan Plateau of China using the method described by Pan^11^,^24^. The strains were preserved on PDA slants and stored at 4 °C.

### Characterisation of endophytic fungus

Genomic DNA from the mycelia (0.4~0.6 g) of fungal endophytes was extracted using the cetyltrimethylammonium bromide (CTAB) method^25^. The ITS1-5.8SrDNA-ITS2 regions were amplified using the universal primers ITS1 and ITS4. The polymerase chain reaction (PCR) was performed using the method described by Pan^24^. The sequencing of the purified PCR products was performed by Tsingke Biological Technology (Beijing, China). The sequences were submitted to the National Center for Biotechnology Information (NCBI) GenBank and analysed by BLAST. MEGA5.1 software was used to estimate the phylogenetic relations of the fungal endophytes. The microscopic characteristics of partial fungal structures that were in doubt after the ITS identification process were observed by optical microscope.

### Cultivation and fermentation of fungi and antioxidant screening

The fungal endophytes were cultivated using the method described by Pan^11^. The filtrate samples were evaluated by identifying their reduction power by FRAP assay and DPPH radical scavenging assay. The details of the test procedures are described below. The FRAP and DPPH values were difficult to compare directly because of their dimensional quantity. Therefore, a fuzzy membership method that had the same value of x in which all the samples had the same membership for 1, was used as described by Chen^23^. The degree of membership with membership function Y was given as Y = (x − x_min_)/(x_max_ − x_min_), x_min_ ≤ x ≤ x_max_. The variables x, x_max_ and x_min_ pertained to the FRAP and DPPH values. In addition, the sum of Y_FRAP_ and Y_DPPH_ was set as the antioxidant activity of the fungal endophytes.

### Preparation of fungal extract

Five strains of fungal endophytes were randomly selected for their good apparent growth and strong antioxidant activity (sum of Y_FRAP_ and Y_DPPH_ > 1). The selected strains, namely 4WBY1, 6WBY3, 7WBY2, WBS019 and WBS020, were fermented under the conditions indicated above. The filtrates were collected and concentrated to up to 20% of the original volume (v/v) in a vacuum rotary evaporator at 50 ± 5 °C. The concentrated filtrates were extracted successively three times with equal volumes of PE, EA, and BA (v/v). The residues were further concentrated to a paste, and extracted with ET. The organic residues were collected, concentrated to dryness and diluted in methanol (PE extraction diluted in PE) to obtain a series of solution concentrations and filtered through a 0.45-μm Millipore filter. The solutions were analysed for their antioxidant activities and chemical components.

### DPPH radical scavenging assay

The DPPH radical scavenging assay was performed as proposed by Makris^26^ with minor modifications. The DPPH reagent was freshly prepared at 0.04 mg·mL^−1^ DPPH in ethyl alcohol solution. Seventy microlitre samples were each added to 200 μL of DPPH solution, mixed and incubated at 27 °C for 10 min. The radical scavenging activity of the tested samples against DPPH was determined by measuring the UV absorbance at 517 nm against methanol blanks using a Multiskan Go microplate reader (MGM) (Thermo Scientific Multiskan^®^ Spectrum, NH, USA). The radical scavenging activity was calculated using the following formula: Scavenging ability for DPPH radicals (%) = (*Abs*_control_ − *Abs*_sample_)/*Abs*_control_ × 100, where *Abs*_control_ was the absorbance of DPPH radical solution without samples. Vc and Trolox were used as the standard antioxidants, and the same holds in the following section.

### Measurement of the total antioxidant capacity (ABTS assay)

A total antioxidant capacity assay was performed with the improved ABTS method proposed by Shan^27^ with slight modifications. Fifteen microlitres of the sample were mixed with 180 μL of ABTS solution. The absorbance was determined by measuring the UV absorbance at 734 nm against deionised water blanks in an MGM after incubation at 37 °C for 30 min in the dark. The absorbance was read when the sample was replaced with methanol as the blank sample. The antioxidant capacity was expressed as the per cent decrease in absorbance at 734 nm, which was calculated using the formula


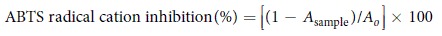


(*A*_*o*_ = absorbance of blank sample, t = 0 min; *A*_sample_ = absorbance of tested sample at the end of the reaction, t = 30 min).

### Measurement of the reduction power (FRAP)

To determine the reducing power of fungal endophytes, the protocol for the FRAP assay was executed as described by Pulido^28^ with slight modifications. Seventy microlitres of the tested sample solution from fungal endophytes was mixed with 200 μL of FRAP reagent and incubated at 37 °C for 4 min. The absorbance was determined at 593 nm against a methanol blank using MGM. In addition, a calibration curve was created using an aqueous solution of ferrous sulphate FeSO_4_ over a concentration series from 25–1100 μM. The FRAP values were expressed on a fresh-weight basis as μmol of ferrous equivalent Fe(II) per L of sample.

### Determination of TPC

TPC from extracts of fungal endophytic fermented filtrates with different organic solvents were determined using the Folin-Ciocalteu method described by Lin^29^ with minor modifications. In brief, the extract sample solutions (1.0 mg·mL^−1^, 2 mL) were diluted to 10 mL with deionised water in 25-mL test tubes. A 0.5 mL volume of Folin-Ciocalteu reagent and 10 mL of 7.5% sodium carbonate (Na_2_CO_3_, w/v) were added. This mixture was incubated at 40 °C for 60 min, diluted with deionised water to 25 mL and mixed. The absorbance was measured at 733 nm against the blank on a spectrophotometer (UV-2450; Shimadzu, Kyoto, Japan, also used for the methods below). The TPC was calculated on the basis of the gallic acid calibration curve, and the TPC levels were determined. The results were expressed as mg of gallic acid equivalents (GAEs) per mg of dry extract.

### Determination of TFC

The total flavonoids were determined using a NaNO_2_-Al(NO_3_)_3_-NaOH colorimetric assay described by Zhu^30^ with minor modifications. In brief, both extract solutions (1.0 mg·mL^−1^, 2 mL) and 4 mL of 60% alcoholic solution (v/v) were accurately added to 25-mL test tubes and 1.0 mL of the NaNO_2_ solution (5%, w/v) was added; the mixture was shaken and left to stand for 6 min. Then, 1.0 mL of the Al(NO_3_)_3_ (10%) solution was added. After 6 min, 10.0 mL of the NaOH solution (10%, w/v) was added, followed by dilution with deionised water to 25 mL. The solution was mixed and incubated at room temperature for 15 min. The reaction mixture absorbance was measured at 507 nm against the blank in the spectrophotometer. The TFC was calculated on the basis of the calibration curve of rutin and the TFC levels were determined. The results were expressed as mg of rutin equivalents (RAEs) per mg of dry extract.

### Determination of TSC

The TSC determination was performed as described by Chen^31^ and Gao^32^ with minor modifications. In brief, the solution containing the extract samples (1.0 mg·mL^−1^, 0.4 mL) and 0.2 mL of 40% alcoholic solution (v/v) were transferred into a 20-mL test tube and mixed. To these solutions, 0.2 mL of freshly prepared 5% (w/v) vanillin-acetic acid solution and 0.8 mL of perchloric acid as the developer were added, mixed and incubated at 70 °C for 15 min. After the mixture cooled to room temperature, 4 mL of EA was added. The reaction mixture absorbance was measured at 554 nm against a blank on the spectrophotometer. The TSC was determined on the basis of an oleanolic acid standard curve, and the TSC levels were determined. The results were expressed as mg of oleanolic acid equivalents (OAEs) per mg of dry extract.

### TLC-Bioautography assay

The TLC-Bioautography assay was performed as described by Thirunavukkarasu, N.^33^ with minor modifications. The PE and EA fractions from the five isolates were subjected to TLC (100 mm × 100 mm) on three silica gel G plates (Qingdao Marine Chemical Factory, Qingdao, China) by parallel analysis. The plates were run in solvent systems containing trichloromethane/toluene/ethanol/formic acid (ratio of 4:4:0.5:0.1, by volume) after equilibration for at least 20 min at 25 °C. Chromatographic spots were visualised using various methods. One plate was visualised with ultraviolet lamps emitting at 365 nm, followed by heating for 3 min at 105 °C after being sprayed with vanillin sulphuric acid (general reagent). The second plate was visualised by ultraviolet lamps emitting at 365 nm after being sprayed with 2% aluminium chloride solution (flavonoid compounds). The third TLC plate was sprayed with 0.04 mg·mL^−1^ DPPH in ethanol, and the presence of yellow stains was indicative of components with antioxidant activity.

### HPLC analysis

An Agilent 1100 series (Agilent, USA) with a Phenomenex Gemini 5 μm C_18_ 110 A column (250 × 4.60 nm, 5 micron, Phenomenex, Torrance, USA) was used as the HPLC system. Samples (5 μL each) were applied to the HPLC system for separation using mobile phases composed of acetonitrile (A) and 0.2% acetic acid aqueous solution (B) at a flow-rate of 1.0 mL/min at 30 °C and the following gradient elution program: 0–12 min, linear gradient 5–40% A; 12–18 min, 40–50% A; 18–22 min, 50–65% A; 22–25 min, 65–95% A; 25–30 min, 95% A; and 30–35 min, 95–5% A. Detection was performed at 280 nm. The quantification of analytes was performed by the external standard curve method. The concentrations of gallic acid, cyanidin-3-O-glucoside, (+)-catechin, chlorogenic acid, caffeic acid, rutin, ferulic acid, phlorizin, icariin, rosmarinic acid, luteolin, and apigenin were determined in the samples. The compounds detected from the largest number fractions were analysed further for correlation with TPC, TFC, TSC, FRAP, DPPH and ABTS activities.

### GC-MS analysis

The PE fractions from fungal endophytes (strain 4WBY1, 6WBY3 and 7WBY2) were subjected to GC/MS analysis using an Agilent Technologies 6890/5973 N GC system series instrument coupled with an HP-5MS capillary column (30 m × 0.25 mm × 0.25 μm). Helium was used as a carrier gas (1 mL·min^−1^). PE fractions (2 μL) were injected onto the GC in splitless mode at an injector temperature of 250 °C. The oven temperature was programmed as follows: 40 °C (4 min hold) −250 °C (3 min hold) at a rate of 5 °C min^−1^, 250–280 °C (6 min hold) at 10 °C min^−1^, and 280–300 °C (3 min hold) at 10 °C min^−1^. The mass spectra were recorded in EI mode (70 eV) in scanning mode (m/z 30–650). The GC/MS peaks were identified by comparing the MS from the published data and matching to a mass spectral library (NIST 11.0).

### Data analysis

All sample antioxidant activities and component determinations were conducted in triplicate, and the results were calculated as the mean ± standard deviation (SD), as in a previous study. Linear correlation coefficients among the TPC, TFC and TSC of fractions extracted from the five isolates and their corresponding antioxidant activities (FRAP, DPPH and ABTS assay) were estimated with Pearson’s correlation coefficient at a 95% confidence interval and paired t tests using SPSS 19.0 software. And the standard curve analysis was calculated using Microsoft Excel 2010.

## Discussion

Fungal endophytes are fungal resources with abundant diversity. All the plants, including non-vascular plants, ferns, conifers and angiosperms, may be symbiotic with fungal endophytes in natural ecosystems^34^. Many previous studies of fungal endophytic diversity have shown that fungal endophytes are multitudinous in host plants such as *Calotropis procera*^35^ and Mexican yew^36^. In the present study, 49 fungal isolates from FUW were distributed across 17 taxa and belonged to 15 genera, including *Cadophora, Plectosphaerella, Paraphoma, Fusarium, Clonostachys (Bionectria*), *Plectosphaerella, Aspergillus, Colletotrichum, Nectria, Thanatephorus, Mucor, Ilyonectria, Cladosporium, Pyrenochaeta* and *Volutella. Fusarium sp*. (11 and 10 isolates of *F. redolens* and *F. tricinctum*, respectively) was the dominant genus. In fact, the fungal endophytes of *Fusarium* have frequently appeared in past studies (e.g., refs 37,38). Abundant and varied bio-activators were produced by the fungal endophytes of *Fusarium*^39^. In spite of the existence of uncultivable fungal endophytes, the result still showed that various fungi were symbiotic with *F. unibracteata* var. *wabuensis* (FUW).

The antioxidant activity of fungal endophytes from medicinal plants is increasingly recognised in natural product research. The antioxidant compounds produced by fungal endophytes likely help the host plant to neutralise ROS. Existing research showed that fungal endophytes can confer effective tolerance to ROS under abiotic stress conditions, and also promote growth via biosynthesis of plant hormones and nutrient acquisition^38^,^40^,^41^,^42^. Not all fungi can live inside plant tissue due to host specificity^43^. Some non-endophytic fungi sources, especially basidiomycetes, also produce numerous antioxidant phenolic compounds^44^. Compared with fungal endophytes, these fungi either cannot live within the tissues of plants or can destroy plant cells and cause oxidative stress (pathogenic fungi). Fungal endophytes and their host plants interact though physical or chemical signals and the former can promote host-plant growth through the production of phytochemicals, including antioxidants, without leading to biotic stress when they invade or live inside host plant tissues^41^. Fungal endophytes from FUW may be also beneficial to their host plant though the production of antioxidant compounds without obvious side effects. To determine the antioxidant activities of these fungal endophytes, we determined the antioxidant activities of the fermentation broth of the isolated fungal endophytes in our study. Most fungal endophytes demonstrated some antioxidant activity. Notably, some fungi, including most *Fusarium spp*. (dominant fungi), had strong activity.

Phenolics, flavonoids and tannins are the main antioxidants in many medicinal plants, such as *Dioscorea bulbifera, Eriobotrya japonica, Tussilago farfara, Ephedra sinica*^17^,^18^,^45^. In addition, many polyphenolics or phenolics, flavonoids, saponins, and others have been isolated from fungal endophytes, such as pestacin^46^. In the current study, the TPC, TFC and TSC results for the extracts of five selected fungal endophytes confirmed that the fungal endophytes from FUW also produce phenolics, flavonoids and saponins. To provide insight into endophytic fungal metabolites, TLC, HPLC and GC/MS analysis methods were used to estimate partial metabolites. TLC-bioautography combined with a new image-processing method has flexibility, simplicity and high-throughput^47^ features for use in determining antioxidant capacity or the active biological compounds such as flavonoids, proteins, phenols^48^. The results of the TLC-bioautography assay revealed the diversity of antioxidants in the metabolites of fungal endophytes, including phenols and flavonoids. In addition, gallic acid, cyanidin-3-O-glucoside, (+)-catechin, chlorogenic acid, caffeic acid, rutin, ferulic acid, phlorizin, icariin, rosmarinic acid, luteolin and apigenin are common phenolics with antioxidative activities. HPLC analysis suggested that most of the above analytes or their derivatives could be produced by fungal endophytes. The GC/MS results also suggested that many phenolics, such as 2,2′-methylenebis(6-tert-butyl-4-methylphenol), phenol, 4-ethyl- and 3,5-di-tert-Butyl-4-hydroxybenzaldehyde, are produced by fungal endophytes. More importantly, all the phenolic compounds have demonstrated antioxidation abilities. For example, 2,2′-methylenebis(6-tert-butyl-4-methylphenol), also known as antioxidant 2246, engages in direct oxidation^49^ and has been used as an antioxidant in the market. Therefore, the results of TLC, HPLC and GC/MS analysis further verified the production of phenolics, flavonoids and saponins with antioxidant behaviour derived from fungal endophytes.

Furthermore, the correlation analysis shows that saponins, phenolics and flavonoids play crucial roles in the antioxidation of fungal endophyte ecological communities from FUW, similar to some plants (such as *Dicliptera roxburghiana*)^50^,^51^,^52^ or single strains (such as *Xylaria* sp. strain YX-28)^53^,^54^,^55^. The TLC, HPLC and GC/MS analysis showed that many common compounds exist in the extracts of multiple isolates, such as the compound with *R*_*f*_ = 0.68 in Fig. 4D (present in the PE and EA fractions from multiple strains), caffeic acid (CA) (present in the EA and BA fractions from four isolates), ferulic acid (FA) (present in the PE or EA fractions from three isolates), phenol and 2,4-bis(1,1-dimethylethyl)- (present in PE fraction from three isolates). CA and FA showed significant correlation (*P* < 0.05) with the antioxidant activities of partial fractions (Table 3). The same or similar antioxidant compounds as those produced by fungi pointed to the antioxidant role of fungal endophytes in FUW. In addition, it should be noted that strains WBS007 and 6WBY3 showed not only good antioxidant activity but also the production of active alkaloids (peimisine, peiminine and imperialine-3β-d-glucoside) in previous studies^11^,^24^. These results further indicate interactions between fungal endophytes and host plants to induce the production of common components.

However, endophytic fungi as important fungi resources will still require further research. For example, when standard antioxidants (caffeic acid and icariin) were cultured with the five isolates, the antioxidant activity (DPPH and ABTS) of the fermentation broth was lower (data not shown). This result showed that multiple antioxidant compounds could be produced, but a superabundance of those compounds exerts feedback control on the fungal metabolite. And there are complicated and various interactions between plant and fungal endophytes, between fungal endophytes, or between metabolic compounds and plant cells or fungi are still unclear. Many publications point to co-evolution and horizontal gene transfer during the long symbiotic process between fungal endophytes and their host plants^11^. It is not known whether horizontal gene transfer occurred at some point during co-evolution or whether fungal endophytes from one plant, such as FUW, share similar biosynthetic pathways with antioxidant compound metabolites. Thus, the fungi from FUW are valuable fungal resources not only to screen active compounds, but also to study the interaction among endophytic fungal cells and biological factors.

## Conclusions

Various fungal endophytes were obtained from FUW bulbs, primarily from the *Fusarium* genus. Most of these fungal endophytes showed antioxidant activity. Moreover, some fungal endophytes from FUW produced multiple phenolic, flavonoid and saponin, including some common active compounds, which played the crucial roles on the antioxidant activities. These fungal endophytes showed the potential to remove ROS from within the host plant and are good resources for obtaining novel or reported antioxidants. However, there are still many unsolved aspects of the metabolic mechanism of antioxidant-compound biosynthetic pathways, including strain improvement to overcome degradation. These fungi are precious materials for further microbial study.

## Additional Information

**How to cite this article**: Pan, F. *et al*. Fungal endophyte-derived *Fritillaria unibracteata* var. *wabuensis*: diversity, antioxidant capacities *in vitro* and relations to phenolic, flavonoid or saponin compounds. *Sci. Rep.* **7**, 42008; doi: 10.1038/srep42008 (2017).

**Publisher's note:** Springer Nature remains neutral with regard to jurisdictional claims in published maps and institutional affiliations.

## Supplementary Material

Supplementary Information

## Figures and Tables

**Table 1:** Culturable endophytic fungal ITS sequences that closely match GenBank sequences.

Isolate number	Accession No. from present study	The source of the closest GenBank match	Location	Accession No.	Identity
1WBY1	KU350694	*Cadophora luteo-olivacea* ICMP:18084	Auckland, New Zealand	HM116747.1	99%
1WBY2	KU350695	*Plectosphaerella cucumerina* strain WM 07.196	Sydney, Australia	KP068972.1	99%
1WBY3	KU350696	*Paraphoma sp. strain P1235*	Frankfurt, Germany	KT268530.1	99%
3WBK1	KU350697	*Fusarium redolens* strain E306	Lanzhou, China	KJ540090.1	99%
3WBY1	KU350700	*Bionectria ochroleuca* isolate DB-5B	Harbin, China	FJ426388.1	99%
3WBY2	KU350701	*Fusarium redolens* strain Sz1_1 H	Szeged, Hungary	KJ584549.1	99%
3WBY3	KU350698	*Plectosphaerella cucumerina* strain WM 07.196	Sydney, Australia	KP068972.1	99%
3WBY4	KU350699	*Fusarium redolens* strain Sz1_1 H	Szeged, Hungary	KJ584549.1	99%
4WBY1	KU350702	*Fusarium sp*. JZ-Z6	Lanzhou, China	EF611091.1	99%
4WBY2	KU350703	*Fusarium tricinctum* isolate GMG105	Lanzhou, China	KJ598871.1	99%
5WBY1	KU350704	*Fusarium sp*. LMG201	Lanzhou, China	KJ598872.1	99%
6WBK1	KU350709	*Nectria sp*. QLF38	Lanzhou, China	FJ025156.1	99%
6WBK2	KU350710	*Nectria sp*. QLF38	Lanzhou, China	FJ025156.1	99%
6WBK3	KU350711	*Fusarium sp*. Zi104	Lanzhou, China	KJ540099.1	99%
6WBY2	KU350705	*Fusarium sp*. LMG201	Lanzhou, China	KJ598872.1	99%
6WBY4	KU350706	*Clonostachys rosea* strain E310	Lanzhou, China	KJ540101.1	99%
6WBY5	KU350707	*Fusarium sp*. LMG201	Lanzhou, China	KJ598872.1	99%
6WBY6	KU350708	*Fusarium sp*. Zi104	Lanzhou, China	KJ540099.1	99%
7WBY1	KU350712	*Fusarium tricinctum* isolate GMG105	Lanzhou, China	KJ598871.1	99%
7WBY2	KU350713	*Fusarium redolens* strain Sz1_1 H	Szeged, Hungary	KJ584549.1	99%
7WBY3	KU350714	*Plectosphaerella cucumerina* strain ASIPC1	Manchester, UK	DQ779781.1	99%
7WBY4	KU350715	*Fusarium sp*. LMG201	Lanzhou, China	KJ598872.1	99%
WBS001	KU350716	*Aspergillus fumigatus* strain A0611	Guangzhou, China	KF577886.1	100%
WBS002	KU350717	*Colletotrichum circinans* strain BBA 67846	Berlin, German	AJ301955.1	99%
WBS003	KU350718	*Nectria haematococca* strain T11	Urumqi, China	KF887043.1	99%
WBS004	KU350719	*Mortierella sp*. T12	Nanjing, China	JF439487.1	99%
WBS005	KU350720	*Colletotrichum circinans* strain BBA 67846	Berlin, Germany	AJ301955.1	99%
WBS008	KU350721	*Thanatephorus cucumeris* isolate XSD-68	Xuzhou, China	EU326212.1	91%
WBS011	KU350722	*Nectria sp*. QLF38	Lanzhou, China	FJ025156.1	100%
WBS012	KU350723	*Mucor hiemalis* isolate B4	Praha, Czech	LN714573.1	99%
WBS014	KU350724	*Fusarium tricinctum* isolate XMJ110	Lanzhou, China	KJ598868.1	99%
WBS015	KU350725	*Fusarium tricinctum* isolate Ffx	Xi’an, China	JX179217.1	99%
WBS016	KU350726	*Ilyonectria sp*. bc_lilb_26_5 h	Durham, USA	KF428351.1	99%
WBS017	KU350727	*Cladosporium sphaerospermum* isolate IJL07	Daegu, South Korea	EU823317.1	99%
WBS018	KU350728	*Fusarium tricinctum* isolate GMG105	Lanzhou, China	KJ598871.1	99%
WBS019	KU350729	*Fusarium tricinctum* strain WS11790	Beijing, China	JX406512.1	99%
WBS020	KU350730	*Fusarium sp*. JZ-Z7	Lanzhou, China	EF611090.1	99%
WBS021	KU350731	*Plectosphaerella cucumerina* MUT < ITA > :5175	Turin, Italia	KT699136.1	99%
WBS022	KU350732	*Aspergillus versicolor* strain SFLs4	Nan Chang, China	HQ443246.1	99%
WBS023	KU350733	*Ascomycota sp*. ZS203	Lanzhou, China	KJ598862.1	99%
WBS024	KU350734	*Nectria sp*. QLF38	Lanzhou, China	FJ025156.1	99%
WBS025	KU350735	*Volutella ciliate*	Beijing, China	GU479901.1	99%
WBS027	KU350736	*Bionectria ochroleuca* strain N. SBA35 (BJTC)	Beijing, China	HQ385971.1	99%
WBS028	KU350737	*Bionectria ochroleuca* strain NG	Harbin, China	FJ238113.1	99%
WBS029	KU350738	*Cladosporium sp*. WZLA007	Qingdao, China	JX029063.1	99%
WBS030	KU350739	*Plectosphaerella cucumerina* strain CSS11769	Beijing, China	JX406529.1	99%
WBS031	KU350740	*Fusarium lateritium* strain: MAFF 235344	Tokyo, Japan	AB587004.1	99%

**Table 2:** Antioxidant screening of fungal endophytes from FUW by FRAP and DPPH assays.

Strains	Reducing power^a^	(%) DPPH ⋅ inhibition^b^	Degree of membership^c^	Strains	Reducing power^a^	(%) DPPH ⋅ inhibition^b^	Degree of membership^c^
1WBY1	143.54 ± 3.07	11.16 ± 14.37	0.12	WBS003	626.31 ± 14.67	49.01 ± 4.73	0.96
1WBY2	360.87 ± 10.21	36.16 ± 1.71	0.58	WBS004	537.98 ± 14.31	17.77 ± 15.58	0.66
1WBY3	553.43 ± 20.06	24.32 ± 0.96	0.65	WBS005	226.27 ± 4.33	60.47 ± 3.93	0.68
2WBY1	663.97 ± 10.65	8.41 ± 1.44	0.61	WBS007	553.87 ± 15.20	34.69 ± 11.20	0.75
3WBK1	723.03 ± 16.77	30.27 ± 2.36	0.88	WBS008	505.41 ± 9.49	21.78 ± 3.22	0.58
3WBY1	553.94 ± 13.50	18.60 ± 2.51	0.60	WBS010	810.04 ± 26.64	79.42 ± 4.86	1.44
3WBY2	634.86 ± 40.17	78.53 ± 0.62	1.26	WBS011	84.60 ± 1.56	11.35 ± 4.73	0.07
3WBY3	616.71 ± 7.09	35.46 ± 5.68	0.82	WBS012	943.90 ± 40.64	95.28 ± 1.28	1.73
3WBY4	—	—	—	WBS013	—	98.86 ± 2.34	0.92
4WBY1※	783.32 ± 31.12	74.18 ± 0.88	1.36	WBS014	703.65 ± 7.27	33.62 ± 4.46	0.89
4WBY2	732.16 ± 25.69	26.71 ± 0.50	0.85	WBS015	603.70 ± 6.12	27.19 ± 3.80	0.73
5WBY1	639.51 ± 13.49	26.44 ± 8.14	0.76	WBS016	515.20 ± 7.94	15.11 ± 2.61	0.52
6WBK1	550.99 ± 7.97	33.80 ± 0.53	0.74	WBS017	—	61.33 ± 2.15	0.55
6WBK2	419.23 ± 30.84	35.65 ± 6.41	0.63	WBS018	924.98 ± 43.86	47.40 ± 5.91	1.24
6WBK3	1086.01 ± 17.32	95.23 ± 2.90	1.86	WBS019※	760.93 ± 5.94	74.35 ± 1.47	1.34
6WBY2	1104.44 ± 25.17	93.01 ± 2.29	1.86	WBS020※	793.82 ± 51.52	82.41 ± 1.01	1.45
6WBY3※	978.97 ± 35.73	82.22 ± 0.32	1.63	WBS021	773.61 ± 21.77	43.43 ± 7.93	1.05
6WBY4	660.76 ± 29.52	6.88 ± 0.14	0.59	WBS022	279.49 ± 11.65	20.00 ± 0.01	0.34
6WBY5	690.46 ± 47.64	60.67 ± 5.52	1.14	WBS023	405.22 ± 14.25	76.04 ± 7.96	1.01
6WBY6	780.42 ± 27.99	83.30 ± 2.17	1.45	WBS024	213.50 ± 18.45	11.91 ± 3.06	0.20
7WBY1	—	—	—	WBS025	168.32 ± 5.48	41.61 ± 4.97	0.44
7WBY2※	824.89 ± 30.18	98.19 ± 7.22	1.64	WBS026	1065.64 ± 23.09	29.03 ± 0.75	1.20
7WBY3	—	—	—	WBS027	741.23 ± 11.55	107.32 ± 8.91	1.64
7WBY4	321.30 ± 5.13	24.57 ± 4.93	0.43	WBS028	496.03 ± 3.06	30.46 ± 3.33	0.66
WBS000	173.57 ± 10.80	37.45 ± 4.90	0.41	WBS029	248.45 ± 16.68	30.86 ± 6.45	0.42
WBS001	684.66 ± 17.55	93.27 ± 3.80	1.45	WBS030	241.45 ± 8.85	17.57 ± 9.86	0.28
WBS002	108.36 ± 4.82	57.47 ± 3.24	0.54	WBS031	599.05 ± 33.79	57.48 ± 49.93	1.30

Note:

^a^Reducing power was determined using the ferric reducing antioxidant power (FRAP) assay (μmol·L^−1^). Expressed as μM Fe(II)/mL filtrate, each value was the mean ± SD (n = 3).

^b^Each value was the mean ± SD (n = 3).

^c^Sum of the degree of membership in Y_FRAP_ and Y_DPPH._

^※^Fungi selected by antioxidant screening.

—Not available.

**Table 3:** Pearson’s correlation coefficient with a 95% confidence interval for all compositions and antioxidant capacities derived via various methods.

	TPC	TFC	TSC	FRAP	DPPH	ABTS	CA	FA
TPC	1							
TFC	0.326	1						
TSC	0.451*	0.394	1					
FRAP	0.005	0.640**	0.094	1				
DPPH	0.588**	0.622**	0.228	0.395	1			
ABTS	0.755**	0.323	0.449*	−0.024	0.633**	1		
CA	−0.628	0.966**	−0.030	0.858	0.629	0.064	1	
FA	0.818	0.975**	0.989**	0.890*	0.888*	0.640	—	1

Note: Significant at the *P* < 0.01 level (^**^), *P* < 0.05 level (^*^); n values of TPC, TFC, TSC, FRAP, DPPH and ABTS were 20, CA and FA were 5. “−” was not calculated.

**Table 4:** Phenolic components from the PE fractions and relative contents according to GC/MS analysis.

NO.	RT/min	Name	Molecular Formula	R_match_ values %	Content※ %
4WBY1					
4	17.12	Phenol, 4-ethyl-	C_8_H_10_O	95	1.35
5	21.29	Phenol, 4-ethyl-2-methoxy-	C_9_H_12_O_2_	90	0.41
9	26.44	Phenol, 2,4-bis(1,1-dimethylethyl)-	C_14_H_22_O	95	0.19
19	37.11	3,5-di-tert-Butyl-4-hydroxyphenylpropionic acid	C_17_H_26_O_3_	97	0.08
6WBY3					
17	26.30	Phenol, 2,4-bis(1,1-dimethylethyl)-	C_14_H_22_O	96	6.77
23	31.89	3,5-di-tert-Butyl-4-hydroxybenzaldehyde	C_15_H_22_O_2_	93	0.87
29	35.46	Benzenepropanoic acid, 3,5-bis(1,1-dimethylethyl)-4-hydroxy-, methyl ester	C_18_H_28_O_3_	96	1.27
32	37.61	3,5-di-tert-Butyl-4-hydroxyphenylpropionic acid	C_17_H_26_O_3_	95	0.48
42	43.88	Phenol, 2,2′-methylenebis[6-(1,1-dimethylethyl)-4-methyl-	C_23_H_32_O_2_	96	0.29
7WBY2					
5	26.36	Butylated Hydroxytoluene	C_15_H_24_O	98	9.71
6	26.89	Phenol, 2,4-bis(1,1-dimethylethyl)-	C_14_H_22_O	97	0.33
24	44.00	Phenol, 2,2′-methylenebis[6-(1,1-dimethylethyl)-4-methyl-	C_23_H_32_O_2_	90	0.12

^※^Content by the area normalisation method.
