# Supplementary material for: Fungal endophyte-derived Fritillaria unibracteata var. wabuensis: diversity, antioxidant capacities in vitro and relations to phenolic, flavonoid or saponin compounds
Source: Sci Rep. 2017 Feb 6;7:42008. doi: 10.1038/srep42008 (PMC5292746; doi:10.1038/srep42008)
Supplement: Supplementary Information [file srep42008-s1.pdf]

Fungal endophytes derived Fritillaria unibracteata var. Wabuensis:

diversity, antioxidant capacities in vitro and relationship with

phenolic, flavonoid or saponin compounds

Feng Pan, Tian-Jiao Su, Shi-Mei Cai, Wei Wu*

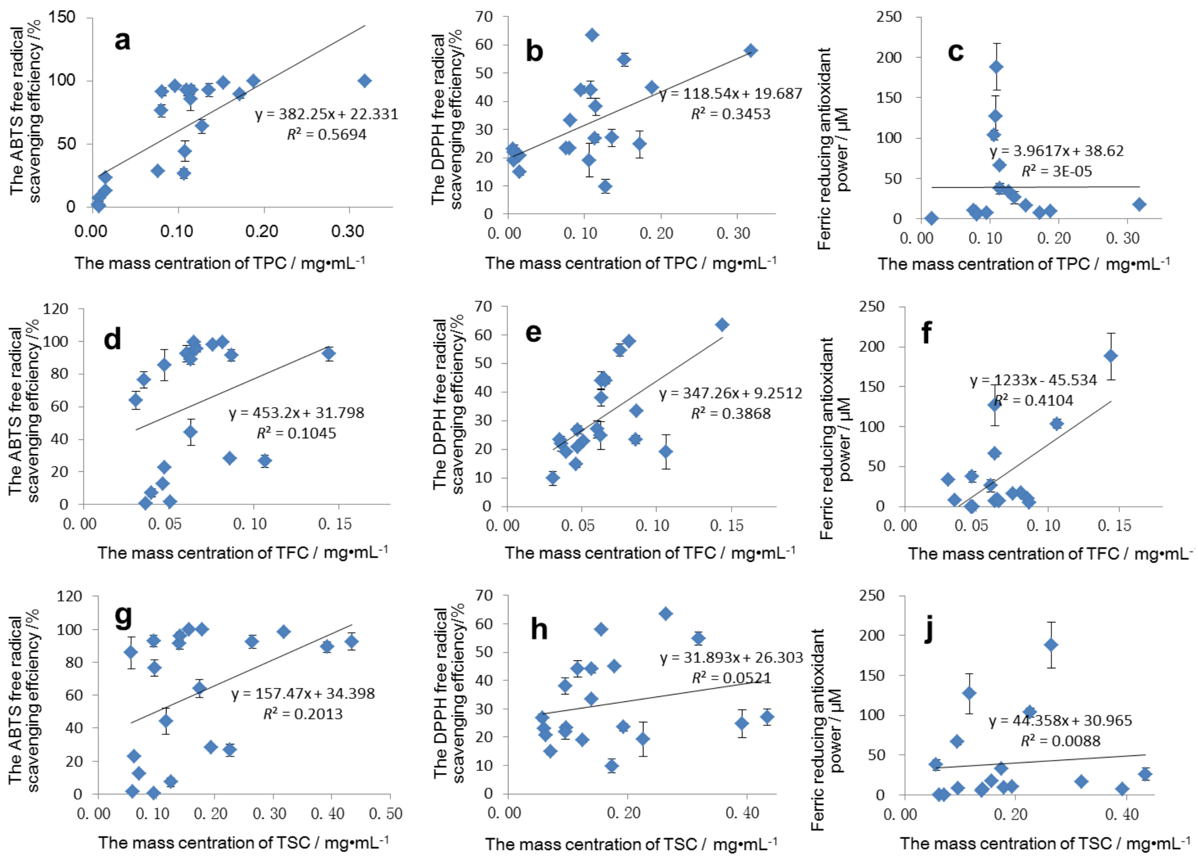

Supplementary Fig. S1: Relationships between antioxidant capacities and compositions (n=20).

a-c: relationships between total phenolic contents (TPC) with total antioxidant capacities (ABTS assay), DPPH free radical scavenging capacities and ferric reducing antioxidant power (FRAP), respectively.

d-f: relationships between total flavonoids content (TFC) with ABTS, DPPH and FRAP capacities, respectively.

g-j: relationships between total saponins content (TSC) with ABTS, DPPH and FRAP capacities, respectively.

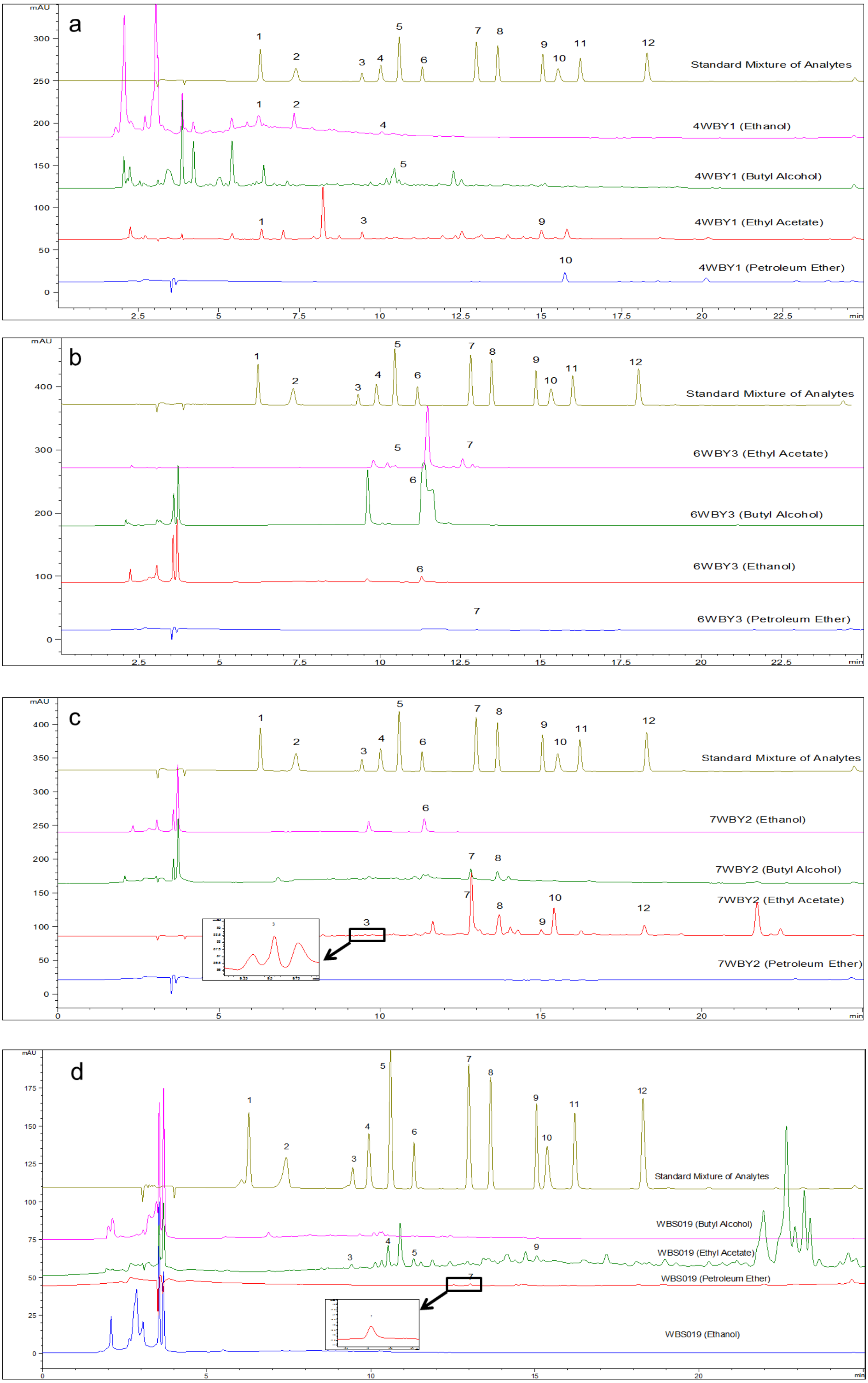
a
Standard Mixture of Analytes
4WBY1 (Ethanol)
4WBY1 (Butyl Alcohol)
4WBY1 (Ethyl Acetate)
4WBY1 (Petroleum Ether)
b
Standard Mixture of Analytes
6WBY3 (Ethyl Acetate)
6WBY3 (Butyl Alcohol)
6WBY3 (Ethanol)
6WBY3 (Petroleum Ether)
c
Standard Mixture of Analytes
7WBY2 (Ethanol)
7WBY2 (Butyl Alcohol)
7WBY2 (Ethyl Acetate)
7WBY2 (Petroleum Ether)
d
Standard Mixture of Analytes
WBS019 (Butyl Alcohol)
WBS019 (Ethyl Acetate)
WBS019 (Petroleum Ether)
WBS019 (Ethanol)
mAU
min

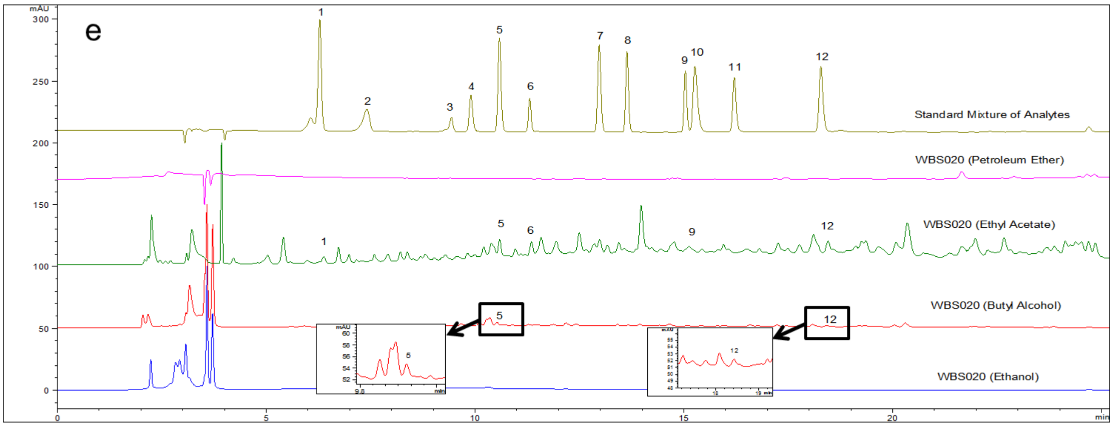

Supplementary Fig. S2: HPLC-DAD chromatogram of n-butyl alcohol (BA), petroleum ether (PE, 30-60 ℃), ethyl acetate (EA) and ethanol absolute (ET) fractions extracted from five representative fungal endophytes and standard mixture analytes.

Peak identification: 1: Gallic acid; 2: Cyanidin-3-O-glucoside; 3: Catechin; 4: Chlorogenic acid; 5: Caffeic acid; 6: Rutinum; 7: Ferulic Acid; 8: Phloridzin; 9: Icariin; 10: Rosmarinic acid; 11: Luteolin; 12: Apigenin;

Supplementary Table S1: The values of IC_{50} (μg/mL) different extract fractions from five strains (4WBY1, 6WBY3, 7WBY2, WBS019 and WBS020) and two standard controls (Vc and Trolox) in scavenging DPPH and ABTS radical scavenging.

	4WBY1	6WBY3	7WBY2	WBS019	WBS020	Vc	Trolox
$IC_{50\ ABTS}$							
BA	1005.13	313.86	589.31	69.92	227.44	42.55	62.95
EA	1524.96	247.66	1738.33	166.33	259.84		
ET	774.73	625.77	417.26	419.39	298.44		
PE	5078.24	6563.56		1087.93	3411.42		
$IC_{50\ DPPH}$							
BA	1018.76	1172.70	3811.51	696.12	1344.46	44.75	32.90
EA	2585.37	834.30	22800.83	1052.87	1757.25		
ET	2697.41	2158.42	3677.68	1808.70	1168.13		
PE	71605.00	17456.67	3528.29	10894.67	9180.00		

BA: n-butyl alcohol extract fraction;
PE : petroleum ether (30-60℃) extract fraction;
EA: ethyl acetate extract fraction;
ET: ethanol absolute extract fraction.

Supplementary Table S2: Chemical composition of the petroleum ether (30–60 ℃) extract from strain 4WBY1.

NO.	RT †	Compounds ‡	Molecular Formula	% Composition
1	15.38	Benzene, 1,2,4,5-tetramethyl-	$C_{10}H_{14}$	0.90
2	16.20	Benzene, 1-ethenyl-4-ethyl-	$C_{10}H_{12}$	0.07
3	16.31	Benzene, 1,2,3,4-tetramethyl-	$C_{10}H_{14}$	0.10
4	17.12	Phenol, 4-ethyl-	$C_8H_{10}O$	1.35
5	21.29	Phenol, 4-ethyl-2-methoxy-	$C_9H_{12}O_2$	0.41
6	22.46	Benzene, 4-ethenyl-1,2-dimethoxy-	$C_{10}H_{12}O_2$	0.33
7	23.02	1-Tetradecene	$C_{14}H_{28}$	0.16
8	26.21	Duroquinone	$C_{10}H_{12}O_2$	1.51
9	26.44	Phenol, 2,4-bis(1,1-dimethylethyl)-	$C_{14}H_{22}O$	0.19
10	27.62	Ethanone, 1-(3,4-dimethoxyphenyl)-	$C_{10}H_{12}O_3$	0.75
11	27.97	7-Hexadecene, (Z)-	$C_{16}H_{32}$	0.59
12	28.11	Hexadecane	$C_{16}H_{34}$	0.14
13	32.48	E-15-Heptadecenal	$C_{17}H_{32}O$	1.41
14	32.56	Octadecane	$C_{18}H_{38}$	0.25
15	32.74	Cyclohexene, 1-methyl-3-(1-methylethenyl)-, (±)-	$C_{10}H_{16}$	1.22
16	35.81	Palmitoleic acid	$C_{16}H_{30}O_2$	1.28
17	36.03	Ethyl 9-hexadecenoate	$C_{18}H_{34}O_2$	2.60
18	36.79	n-Hexadecanoic acid	$C_{16}H_{32}O_2$	5.89
19	37.11	3,5-di-tert-Butyl-4-hydroxyphenylpropionic acid	$C_{17}H_{26}O_3$	0.08
20	37.58	Tetradecanoic acid	$C_{14}H_{28}O_2$	0.21
21	39.00	Paclobutrazol	$C_{15}H_{20}ClN_3O$	0.20
22	39.79	Oleic Acid	$C_{18}H_{34}O_2$	9.72
23	39.93	9-Octadecenoic acid, (E)-	$C_{18}H_{34}O_2$	0.51
24	40.13	Octadecanoic acid	$C_{18}H_{36}O_2$	2.59
25	40.63	Ethanone, 2-(1H-imidazo[4,5-b]pyridin-2-yl)-1-(4-morpholyl)-	$C_{12}H_{14}N_4O_2S$	0.17
26	41.91	9,12,15-Octadecatrienoic acid, ethyl ester, (Z,Z,Z)-	$C_{20}H_{34}O_2$	0.23
27	42.32	Quinoxaline, 6-(3-nitrobenzylidenamino)-	$C_{15}H_{10}N_4O_2$	0.13
28	45.73	Phthalic acid, di(2-propylpentyl) ester	$C_{24}H_{38}O_4$	0.09
29	55.33	Solanidan-3-one	$C_{27}H_{43}NO$	0.24

† RT: Retention time (as min)

‡ Compounds listed in order of retention time.

Supplementary Table S3: Chemical composition of the petroleum ether (30–60 ℃) extract from strain 6WBY3.

NO.	RT †	Compounds ‡	Molecular Formula	% Composition
1	14.26	Benzene, 1-ethyl-2,4-dimethyl-	$C_{10}H_{14}$	0.07
2	15.30	Benzene, 1,2,3,4-tetramethyl-	$C_{10}H_{14}$	0.42
3	15.88	1H-Indene, 2,3-dihydro-4-methyl-	$C_{10}H_{12}$	0.09
4	16.25	Benzene, 1,2,4,5-tetramethyl-	$C_{10}H_{14}$	0.31
5	17.21	Naphthalene	$C_{10}H_{8}$	0.05
6	19.09	Hexanedioic acid, dimethyl ester	$C_{8}H_{14}O_{4}$	0.06
7	20.58	Tridecane	$C_{13}H_{28}$	0.04
8	22.61	Dodecane, 2,6,10-trimethyl-	$C_{15}H_{32}$	0.06
9	23.07	2-Tetradecene, (E)-	$C_{14}H_{28}$	0.57
10	23.30	Tetradecane	$C_{14}H_{30}$	0.62
11	23.39	Longifolene	$C_{15}H_{24}$	0.11
12	24.81	Heptadecane, 2,6,10,14-tetramethyl-	$C_{21}H_{44}$	0.20
13	24.92	2,5-Cyclohexadiene-1,4-dione, 2,6-bis(1,1-dimethylethyl)-	$C_{14}H_{20}O_{2}$	0.05
14	25.00	Tetradecane, 3-methyl-	$C_{15}H_{32}$	0.05
15	25.12	Naphthalene, 2-bromo-	$C_{10}H_{7}Br$	0.04
16	25.80	Pentadecane	$C_{15}H_{32}$	0.93
17	26.30	Phenol, 2,4-bis(1,1-dimethylethyl)-	$C_{14}H_{22}O$	6.77
18	26.95	Cyclotetradecane	$C_{14}H_{28}$	0.18
19	28.17	1-Tricosene	$C_{23}H_{46}$	3.01
20	28.35	Hexadecane	$C_{16}H_{34}$	1.57
21	29.45	2-Bromo dodecane	$C_{12}H_{25}Br$	1.48
22	30.58	Pentadecane, 2,6,10,14-tetramethyl-	$C_{19}H_{40}$	2.15
23	31.89	3,5-di-tert-Butyl-4-hydroxybenzaldehyde	$C_{15}H_{22}O_{2}$	0.87
24	32.58	1-Octadecanol	$C_{18}H_{38}O$	7.33
25	32.85	Octadecane	$C_{18}H_{38}$	1.75
26	32.93	Hexadecane, 2,6,10,14-tetramethyl-	$C_{20}H_{42}$	0.59
27	34.06	Phthalic acid, butyl tetradecyl ester	$C_{26}H_{42}O_{4}$	2.41
28	35.14	7,9-Di-tert-butyl-1-oxaspiro(4,5)deca-6,9-diene-2,8-dione	$C_{17}H_{24}O_{3}$	3.07
29	35.46	Benzenepropanoic acid, 3,5-bis(1,1-dimethylethyl)-4-hydroxy-, methyl ester	$C_{18}H_{28}O_{3}$	1.27
30	35.94	1,2-Benzenedicarboxylic acid, butyl 8-methylnonyl ester	$C_{22}H_{34}O_{4}$	3.15
31	36.87	n-Hexadecanoic acid	$C_{16}H_{32}O_{2}$	19.02
32	37.61	3,5-di-tert-Butyl-4-hydroxyphenylpropionic acid	$C_{17}H_{26}O_{3}$	0.48
33	38.35	Ethanol, 2-(tetradecyloxy)-	$C_{16}H_{34}O_{2}$	0.86

34	39.08	13-Tetradecen-1-ol acetate	$C_{16}H_{30}O_2$	0.40
35	39.98	1-Nonadecene	$C_{19}H_{38}$	10.04
36	40.76	Isopropyl stearate	$C_{21}H_{42}O_2$	0.89
37	41.28	Eicosane	$C_{20}H_{42}$	0.07
38	41.88	Tricosane	$C_{24}H_{50}$	0.47
39	42.32	17-Pentatriacontene	$C_{35}H_{70}$	0.05
40	42.99	Docosane	$C_{22}H_{46}$	0.16
41	43.59	Z-5-Nonadecene	$C_{19}H_{38}$	3.56
42	43.88	Phenol, 2,2'-methylenebis[6-(1,1-dimethylethyl)-4-methyl-	$C_{23}H_{32}O_2$	0.29
43	45.01	Heptadecane	$C_{17}H_{36}$	0.27
44	45.09	Tetracosane	$C_{24}H_{50}$	0.09
45	45.76	Bis(2-ethylhexyl) phthalate	$C_{24}H_{38}O_4$	0.51
46	46.55	1-Docosene	$C_{22}H_{44}$	1.47
47	47.74	1-Tricosene	$C_{23}H_{46}$	0.05
48	48.25	Heptacosane	$C_{27}H_{56}$	0.10
49	51.59	Nonacosane	$C_{29}H_{60}O$	0.07
50	55.91	Dotriacontyl heptafluorobutyrate	$C_{36}H_{65}F_7O_2$	0.28

† RT: Retention time (as min)

‡ Compounds listed in order of retention time.

Supplementary Table S4: Chemical composition of the petroleum ether (30–60 ℃) extract from strain 7WBY2.

NO.	RT †	Compounds ‡	Molecular Formula	% Composition
1	15.40	Benzene, 1,2,3,5-tetramethyl-	$C_{10}H_{14}$	0.12
2	16.50	Pyridine, 3-butyl-	$C_9H_{13}N$	0.26
3	23.06	2-Tetradecene,(E)-	$C_{14}H_{28}$	0.23
4	23.26	Tetradecane	$C_{14}H_{30}$	0.17
5	26.36	Butylated Hydroxytoluene	$C_{15}H_{24}O$	9.71
6	26.89	Phenol, 2,4-bis(1,1-dimethylethyl)-	$C_{14}H_{22}O$	0.33
7	27.39	Pentadecane,2-methyl-	$C_{16}H_{34}$	0.13
8	28.48	7-Hexadecene, (Z)-	$C_{16}H_{32}$	7.45
9	29.73	Hexadecane,2-methyl-	$C_{17}H_{36}$	0.29
10	30.92	Pentadecane, 2,6,10,14-tetramethyl-	$C_{19}H_{40}$	4.76
11	32.89	1-Octadecene	$C_{18}H_{36}$	2.72
12	33.34	Hexadecane, 2,6,10,14-tetramethyl-	$C_{20}H_{42}$	0.90
13	34.57	Phthalic acid, isobutyl octyl ester	$C_{20}H_{30}O_4$	2.69
14	34.75	Heptadecane,3-methyl-	$C_{18}H_{38}$	0.21
15	35.60	Phenanthrene-1-methyl-	$C_{15}H_{12}$	0.62
16	35.42	Pentadecanoic acid, 14-methyl-, methyl ester	$C_{17}H_{34}O_2$	1.00
17	36.30	1,2-Benzenedicarboxylic acid, butyl 8-methylnonyl ester	$C_{22}H_{34}O$	3.76
18	36.79	E-15-Heptadecenal	$C_{17}H_{32}O$	5.10
19	38.01	1-Eicosene	$C_{20}H_{40}$	0.35
20	38.51	Cyclotetradecane	$C_{14}H_{28}$	2.38
21	40.31	1-Nonadecene	$C_{19}H_{38}$	5.29
22	42.03	Cyclotetracosane	$C_{24}H_{48}$	2.07
23	43.57	1-Docosene	$C_{22}H_{44}$	2.01
24	44.00	Phenol, 2,2'-methylenebis[6-(1,1-dimethylethyl)-4-methyl-	$C_{23}H_{32}O_2$	0.12
25	45.12	4,5-Dihydrobenzo[1,2-c:3,4-c']bis[1,2,5]oxadiazole-1,6-dioxide	$C_6H_4N_4O_4$	0.15
26	45.87	Bis(2-ethylhexyl) phthalate	$C_{24}H_{38}O_4$	0.73
27	48.76	10-Heneicosene(c,t)	$C_{21}H_{42}$	3.79

† RT: Retention time (as min)

‡ Compounds listed in order of retention time.
